# Effects of Context and Virtual Reality Environments on the Wine Tasting Experience, Acceptability, and Emotional Responses of Consumers

**DOI:** 10.3390/foods9020191

**Published:** 2020-02-14

**Authors:** Damir D. Torrico, Yitao Han, Chetan Sharma, Sigfredo Fuentes, Claudia Gonzalez Viejo, Frank R. Dunshea

**Affiliations:** 1 Faculty of Veterinary and Agricultural Sciences, School of Agriculture and Food, The University of Melbourne, Parkville VIC 3010, Australia; yitaoh1@student.unimelb.edu.au (Y.H.); sigfredo.fuentes@unimelb.edu.au (S.F.); claudia.gonzalez@unimelb.edu.au (C.G.V.); fdunshea@unimelb.edu.au (F.R.D.); 2 Department of Wine, Food and Molecular Biosciences, Faculty of Agriculture and Life Sciences, Lincoln University, Lincoln 7647, New Zealand; Chetan.Sharma@lincoln.ac.nz

**Keywords:** virtual reality, acceptability, Cabernet Sauvignon, wine, context, emotions

## Abstract

Wine tasting is a multidimensional experience that includes contextual information from tasting environments. Formal sensory tastings are limited by the use of booths that lack ecological validity and engagement. Virtual reality (VR) can overcome this limitation by simulating different environmental contexts. Perception, sensory acceptability, and emotional responses of a Cabernet Sauvignon wine under traditional sensory booths, contextual environments, and VR simulations were evaluated and compared. Participants (*N* = 53) performed evaluations under five conditions: (1) traditional booths, (2) bright-restaurant (real environment with bright lights), (3) dark-restaurant (real environment with dimly lit candles), (4) bright-VR (VR restaurant with bright lights), and (5) dark-VR (VR restaurant with dimly lit candles). Participants rated the acceptability of aroma, sweetness, acidity, astringency, mouthfeel, aftertaste, and overall liking (9-point hedonic scale), and intensities of sweetness, acidity, and astringency (15-point unstructured line-scale). Results showed that context (booths, real, or VR) affected the perception of the wine’s floral aroma (dark-VR = 8.6 vs. booths = 7.5). Liking of the sensory attributes did not change under different environmental conditions. Emotional responses under bright-VR were associated with “free”, “glad”, and “enthusiastic”; however, under traditional booths, they were related to “polite” and “secure”. “Nostalgic” and “daring” were associated with dark-VR. VR can be used to understand contextual effects on consumer perceptions.

## 1. Introduction

The globalization of markets, an increase in per capita income (especially in developing countries), and eventually increased spending power has allowed more consumers to access wine products from around the world [1]. With the increasing consumers’ wine demand and increased competition, the production of higher quality and more acceptable wines is one of the biggest challenges for the wine industry to remain relevant in the market [2]. Sensory analysis of wines is an essential and critical component of quality control [3,4]. The chemical composition measured by instrumental analysis can provide valuable and reliable information to assess the quality of wine products; however, the consumers’ assessment is a critical decision-making tool to test the success of the product in the marketplace [1,2]. Sensory analysis, which relies on the sight, smell, taste, touch, and hearing senses to determine the intrinsic properties of wine products provides a multidimensional response that is closely related to the tasting experience of consumers. From the vineyards (grape quality) to the final wine products that are being consumed on various occasions, sensory analysis is an important tool in the manufacturing chain of wine production and commercialization [5].

Traditional wine assessment depends on the tasting abilities of a panel of experts who rate the quality of wines using an attribute-based grading system [2]. However, this method only uses a small number of scores that might not explain the acceptability of the product entirely. On the other hand, standard consumers’ sensory evaluation relies on using isolated booths for removing external environmental factors, including odors and noises that can produce biases in responses [6,7]. However, these controlled environments lack ecological validity because they do not offer “real-world” conditions for replicating the authentic tasting experience of consumers [8]. Sensory laboratories can be considered unfamiliar environments for the consumption of foods and beverages, where consumers are separated in booths and isolated from external stimuli. In reality, consumption of foods and beverages is influenced by the environmental factors that stimulate the senses of consumers [9,10,11]. The contextual elements are complex and variable, including several sensory stimuli, which constitute the background information of the tasting experience. The different external factors create complex contextual cues that are essential for the formation of subsequent consumers’ behaviors, expectations, and hedonic evaluations [12,13,14,15]. Besides the sensory characteristics of products, external factors surrounding consumers (environmental and social interaction) can also affect the acceptability and emotional responses [16]. 

The lack of active consumers’ engagement in the sensory and hedonic evaluations can reduce the prediction rates of product choice, which leads to a higher probability of failure in the research and development of new products in the food and beverage industry [8]. The primary purpose of using a sensory laboratory is to collect responses generated by the senses of participants with the elimination of the influences produced by external factors [17]. Compared to traditional sensory laboratory setups, “real-world” environments have multiple external variables that are difficult to quantify and control. For instance, Dorado, Chaya, Tarrega, and Hort [16] stated that the contextual information could affect the emotional responses of consumers towards beer products, but this effect was not significant for liking. Sester et al. [18] found that different contexts, produced by the simulation of two bar environments using immersive technology (bars were decorated with cold and warm tone lights with different background pictures), had a significant effect on consumers’ choices of drinks. Nijman et al. [19] found that consumers were able to discriminate lager and ale beers better using a real bar as a testing environment compared to that of traditional sensory booths. However, conducting sensory research in external locations is challenging because it can be generally time-consuming and expensive [20].

The creation of a simulated virtual environment within the controllable laboratory setup for the sensory evaluation of wine products could be a step closer to pursue ecological validity at a relatively lower cost. Virtual reality (VR) technology, which combines a series of interactive computer-generated images or videos that link users’ minds and sensory systems, can be applied to simulate environments similar to the “real world” [21]. The virtual environment provided by the VR technology usually is generated by using VR headsets, which can offer visual and auditory stimuli with the advantage of being easy to operate [22]. VR technology can generate virtual scenes throughout dynamic environments that make participants feel engaged by using stereoscopic displays and sensing technology. Moreover, compared with the “real-world” environment, the conditions of VR environments are relatively controllable, and the environments are easy to replicate. There is a growing interest in the scientific community on studying the effects of VR on sensory and consumer sciences [23,24,25]. Regarding the use of real surroundings, Hannum et al. [26] evaluated the effect of three contextual environments (traditional sensory booths, an immersive wine bar, and an actual wine bar) on the acceptability and purchase intent of consumers toward wine samples. Although they found that the environment had a marginal effect on acceptability, individual consumers’ behaviors changed depending on the environment that was used. There is still very little information on how the VR environments perform against “real-world” contexts. Therefore, this study evaluated the perception, sensory acceptability, and emotional responses of a Cabernet Sauvignon wine under different conditions, including traditional sensory booths, “real-word” contextual environments, and VR simulations with associated hardware.

## 2. Materials and Methods

### 2.1. Participants

The research protocol for this study was listed as a minimal risk with the ethics approval 1543704.2 obtained in February 2017 by the Human Ethics Advisory Group (HEAG) of the Faculty of Veterinary and Agricultural Science at The University of Melbourne, Australia. A total of *N* = 53 participants (12 males and 41 females) with ages of 35 ± 10 years old were recruited from a pool of students and staff belonging to The University of Melbourne to participate in this study. A power analysis was performed to verify that the number of participants (*N*) used in the present study was adequate (*Power* = 0.93). All participants were untrained and reportedly not allergic to any food product. Participants who consumed wine products at least once per month were pre-selected for the sensory sessions. All participants were asked to sign a consent form approved by the Human Ethics Advisory Group (The University of Melbourne) before tasting the wine. Sensory sessions were held at the sensory laboratory facilities of The University of Melbourne, Australia, which is composed of 20 sensory booths and focus group rooms. Five sensory sessions (traditional booths, bright-restaurant, dark-restaurant, bright-VR, and dark-VR) were conducted on five different days. The order of the sessions was randomized within each participant. The duration of one sensory session was approximately 20 to 30 min for each participant. At the end of all five sessions, participants were rewarded with a $10 gift card.

### 2.2. Stimulus

A descriptive panel (*N* = 8) from The University of Melbourne was used to select the Cabernet Sauvignon wine for this study. Six red wines were classified as high, medium, and low according to their average quality scores (100 points). A medium-quality (*score* = 76.25) Cabernet Sauvignon wine (Phoenix, Penley Estate, Coonawarra, SA, Australia) was used as the product stimulus for this experiment. Only one medium-quality red wine was selected because the focus of this experiment was to measure the effects of the context and environment on the taste perception of the wine. Bottles of 750 cc wines from the same batch were purchased from a local grocery store and kept at 16 °C. Five hours before the sensory sessions, the bottles of wines were transferred to the tasting room to reach room temperature (25 °C). Wine bottles were wrapped with aluminum foil to hide any packaging cues that can bias the responses of participants. A total of 15 mL of wine was poured in standard wine clear glasses (international standard wine tasting glasses, Luigi Bormioli International Organization for Standardization (ISO) wine tasting glasses with a rim diameter of 46 mm, height of 155 mm, and a total volume of 215 mL). For each environment, participants received two wine samples with different three-digit random codes from the same bottle (duplicates). This was done to avoid positional biases, logical errors, and pattern effects that can lead to guessing the nature of the samples. The purpose of this test was to measure the effect of the context and environment on consumers’ responses having samples with the same taste.

### 2.3. Sensory Procedure

**Figure 1:**
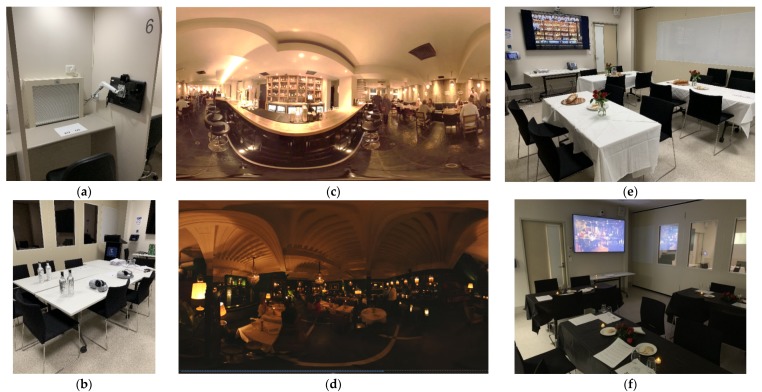
Experimental settings ^1^ for the sensory evaluation of the wine. ^1^ (**a**) Traditional sensory booths; (**b**) VR set-up; (**c**) bright restaurant VR environment; (**d**) dark restaurant VR environment; (**e**) bright real restaurant environment; and (**f**) dark real restaurant environment.

At the beginning of the tasting sessions, instructions were provided to each participant explaining the experimental procedures, including the proper operation and wearing of VR headsets, as well as information about how to fill out the paper ballots. After a brief explanation of the test procedures, participants who were willing to continue with the sensory test signed the consent form. The environments in this study included (1) traditional booths, (2) bright-restaurant (real environment illuminated with bright lights), (3) dark-restaurant (real environment illuminated with dimly lit candles), (4) bright-VR (VR restaurant illuminated with bright lights), and (5) dark-VR (VR restaurant illuminated with dimly lit candles) (Figure 1). In the VR sessions, participants were instructed to wear the VR headsets and taste the wine with the help of the instructor in the room. For the immersive (real) and traditional booth sessions, participants were seated in the tables and had the samples ready in front of them for tasting. Participants tasted two wine samples (using the same wine) in each session. The presentation of the samples was randomized, and a sequential monadic sample order was used within each participant. In the VR sessions, participants were instructed to take VR headsets off and start answering questions once they finished tasting. This process was repeated for each participant until the tasting of all samples was completed under each VR setting. In the immersive (real) and traditional booth sessions, participants were instructed to taste the wine samples from left to right and answer the questions in the paper ballots. In the paper ballots, participants were asked to rate the acceptability of the floral aroma, fruity aroma, sweetness, acidity, mouthfeel-body, astringency, aftertaste, and overall liking of the red wine sample using the nine-point hedonic scale (1 = disliked extremely, 5 = neither liked nor disliked, 9 = liked extremely). The intensities of floral aroma, sweetness, acidity, and astringency were evaluated using a 15 cm unstructured line scale. Sweetness, acidity, astringency, and body were also assessed using a just-about-right scale (JAR; for sweetness, acidity, and astringency: 1 = too little, 2 = just about right, 3 = too much; for body: 1 = light, 2 = medium, 3 = full). Purchase intent (question: “If this product is available on the market, will you buy it?”) of the wine samples was evaluated using a binomial scale (1 = yes, 2 = no). No re-tasting of samples was allowed in the current experiment, and all the collected responses were memory-based.

To assess the emotional responses elicited by the wine tasting experience in the different environments, a check-all-that-apply (CATA) procedure was applied using a list of 33 emotional terms (adventurous, pleased, satisfied, pleasant, active, secure, affectionate, warm, calm, bored, energetic, disgusted, enthusiastic, worried, free, aggressive, friendly, daring, glad, eager, good, guilty, happy, polite, interested, steady, joyful, understanding, loving, wild, merry, nostalgic, and peaceful).

These emotion terms were pre-selected from a list containing 48 emotional terms obtained from previous studies [27,28] to cover two-dimensional affective spaces (valence and arousal), according to Bradley and Lang [29]. Participants used water and unsalted crackers to cleanse their palate in between wine samples.

### 2.4. Test Environments

As shown in Figure 1, five test environments (traditional booths, bright-restaurant, dark-restaurant, bright-VR, and dark-VR) were used for the tasting experiences of the wine. Traditional booths (traditional sensory evaluation environment) consisted of isolated individual booths located in the sensory laboratory facilities at the University of Melbourne, Australia (Figure 1a). The dimensions of the sensory testing booths were 1.5 m (width) × 2.1 m (height) with a worktop used for placing samples and questionnaires. The sensory booths were illuminated with light emitting diode (LED) light (configured with white color; RGB = 255, 255, 255). The temperature of the sensory room was set at 25 °C. The VR environments used in this study (restaurants with bright and dark ambient) were both obtained from YouTube (Google LLC, San Bruno, CA, USA), and were selected from a pool of 10 VR environments (YouTube) in preliminary focus group discussions (*N* = 6) (bright-VR contextual environment (https://www.youtube.com/watch?v=y8iqpLN-YIE), and dark-VR contextual environment (https://www.youtube.com/watch?v=2zYWdAqmxBw)) The “real” restaurant environments were assembled to create two restaurant conditions for this study (bright and dark conditions). Before the testing session, wine samples coded with three-digit random numbers and questionnaires (paper ballots) were placed on the table for the participants. 

Consumer tests in the “real” restaurant contexts were conducted in two separate rooms having bright and dark environments (Figure 1e,f, respectively). In the bright-restaurant environment, participants were seated in restaurant-type tables that were decorated with a vase with flowers and a wooden table with bread. In the bright-restaurant room, there was a 60 inch TV screen (Samsung, Seoul, Korea) that displayed a photo of a wine cabinet with a wide variety of wines. In the dark-restaurant environment, participants were seated in a dark room that was illuminated by artificial LED tea-lights (Kmart, Melbourne, VIC, Australia), and decorated with flowers and plates. In the dark-restaurant room, there was also a 60 inch TV screen that displayed a photo of a dark-restaurant room.

The consumer test sessions under the VR environments were conducted in a private and isolated focus-group room (Figure 1b). The VR environments were generated by an Oculus Go all-in-one VR headset with a controller (Facebook Technologies, LLC, Menlo Park, CA, USA), which provided the dynamic visual scenarios (Figure 1c,d). Throughout the VR testing process, a testing supervisor was always present to help the subjects wear the VR headsets. After the tasting of each wine sample, participants were asked to remove their VR headsets and answer the questions on the paper ballots. The bright-restaurant VR environment was a restaurant-type room illuminated with bright lights. The subjects in this environment tasted the wines in a simulated restaurant bar, facing a cabinet that has a wide variety of wines (Figure 1c). During the tasting experience, participants were able to see, hear, and feel the activities of other persons dining in the restaurant. The background noise for this environment included low-pitched conversations from other persons in the restaurant. On the other hand, the dark-restaurant VR was placed in a restaurant illuminated with dim lights with no background music. The restaurant had several tables with seated persons that were having low-pitched conversations. Table lamps with yellow lights were placed in several places in the restaurant. Participants in this VR environment were placed in one table at the corner of the restaurant (Figure 1d).

### 2.5. Statistical Analysis

A two-way ANOVA (environment × order) with a generalized linear model (GLM) and a post-hoc Tukey’s Honest Significant Difference (HSD) test were used to asses significant differences among the evaluated environments (traditional booths, bright-restaurant, dark-restaurant, bright-VR, and dark-VR) for the hedonic ratings and intensity scores of the Cabernet Sauvignon wine. The order effect was included in the ANOVA model to test whether the position of the samples was a significant factor when tasting the wine. A penalty test on the JAR data was performed to determine the effects of sensory attributes on the hedonic liking. Mean drops for the “too much” and “too little” scores were calculated (differences between the liking mean for the JAR level minus the “too much” or “too little” levels). For the CATA frequency data, correspondence analysis and principal coordinate analysis were used to assess the differences among the evaluated environments relative to the selection of the emotion terms and overall liking levels. For the purchase intent, the Cochran’s Q test and simultaneous confidence intervals testing were used for multiple comparisons. A principal component analysis (PCA) was applied to interpret relationships between the hedonic ratings and intensity scores of the wine in different environments. A product-attribute biplot was used for the illustration of the PCA. Data were analyzed at α = 0.05 using the XLSTAT Statistical Software version 2017 (Addinsoft, New York, NY, USA). All data were reported as mean values with standard errors.

## 3. Results

### 3.1. Sensory Responses to the Wine Sample in Different Environments

Results of the analysis of variance (ANOVA) for the different sensory parameters are shown in Table 1 (acceptability and intensity). For the acceptability responses (floral aroma, fruity aroma, sweetness, acidity, mouthfeel astringency, body, aftertaste, and overall liking), none of the main effects (environment nor order) were significant (*p* ≥ 0.05) in the ANOVA models. The interaction (environment*order) effect was only significant (*p* < 0.05) for the fruity aroma attribute on the model of acceptability. For the intensity parameters (floral aroma, sweetness, acidity, and astringency), the type of environment effect was only significant (*p* < 0.05) for the floral aroma and astringency parameters. The interaction effect (environment*order) was only significant (*p* < 0.05) for the acidity and astringency intensity parameters. The order main effect was not a significant factor (*p* ≥ 0.05) for neither the acceptability nor the intensity parameters; therefore, the means of the two served samples could be pooled for the *post-hoc* means comparison analysis.

Table 2 shows the mean acceptability scores of the red wine sample in each environmental condition (traditional booths, bright-restaurant, dark-restaurant, bright-VR, and dark-VR). For the aroma acceptability attributes, the floral aroma scores for all the environments were not significantly different (*p* ≥ 0.05) among them, ranging from 5.65 (dark-VR) to 5.94 (dark-restaurant). On the other hand, the acceptability score of the fruity aroma of the wine sample in the dark-restaurant environment was significantly higher (*p* < 0.05) compared to that of the wine sample in the traditional booths (5.99 vs. 5.65, respectively). No significant differences (*p* ≥ 0.05) were found among the environments in the taste acceptability parameters (sweetness, acidity, astringency, body, and aftertaste) and overall liking of the wine sample. The mean intensity scores of the wine sample in each environment for the floral aroma, sweetness, acidity, and astringency are also shown in Table 2. The real dark-restaurant environment had a significantly (*p* < 0.05) higher floral aroma intensity score compared to those values of the traditional booths and the VR environments (bright and dark; 8.61 vs. 7.45–7.96). On the other hand, the real bright-restaurant had a significantly (*p* < 0.05) higher floral aroma intensity score compared to the value of the traditional booths (8.14 vs. 7.45, respectively), but was not significantly (*p* ≥ 0.05) different compared to the VR environments (bright and dark). Opposite results were observed for the astringency intensity attribute, in which the real dark-restaurant environment had a significantly (*p* < 0.05) lower score compared to that of the traditional booths and the dark-VR environment (7.57 vs. 8.34–8.39, respectively; Table 2). For the sweetness and acidity intensity attributes, no significant differences (*p* ≥ 0.05) were found among the environments.

**Figure 2:**
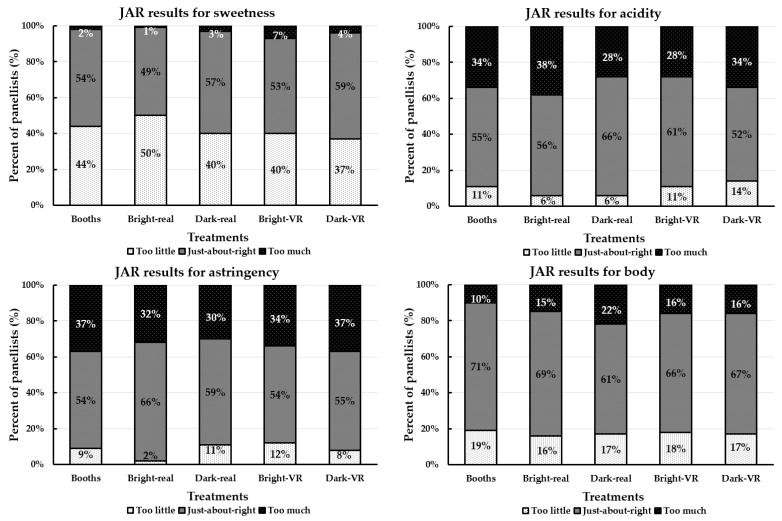
Selection frequencies (%) of just-about-right (JAR) results for the sweetness, acidity, astringency, and body of the wine sample in different environments ^1^. ^1^ Booths = traditional sensory booths, Bright-real = bright restaurant real environment, Dark-real = dark restaurant real environment, Bright-VR = bright restaurant virtual reality (VR) environment, and Dark-VR = dark restaurant VR environment.

The frequency distribution (%) of participants’ responses for the intensities of sweetness, acidity, astringency, and body of the wine sample in each environment using the just-about-right (JAR) scale is shown in Figure 2. In general, the wine sample was considered to be “just-about-right” (49%–59%) and “too little” (37%–50%) in the sweetness for all tested environments in this study. Moreover, participants considered the wine sample to be “just-about-right” (52%–66%) and “too much” (28%–38%) in acidity and astringency for all environments. For the body of the wine sample, participants rated this attribute as “just-about-right” (61%–71%) for all the environments (Figure 2). The penalty analysis using the JAR data is shown in Table 3. In general, the wine sample for all the environments was considered to be “too little” in sweetness (*mean drop* = 1.25–1.99; *p* < 0.05) except for the dark-VR environment, in which the mean drop was not significant in the overall liking (0.40; *p* ≥ 0.05). 

Participants penalized the wine sample in all the environments for being “too much” in acidity (*mean drop* = 1.17–1.94) and astringency (1.02–1.94), but they did not penalize the body attribute (*p* ≥ 0.05; Table 3). Moreover, the purchase intent values of the wine samples in all the environments were not significantly (*p* ≥ 0.05) different (42%–45%; data not shown).

### 3.2. Emotions and Multivariate Analysis of the Wine Sample in Different Environments

**Figure 3:**
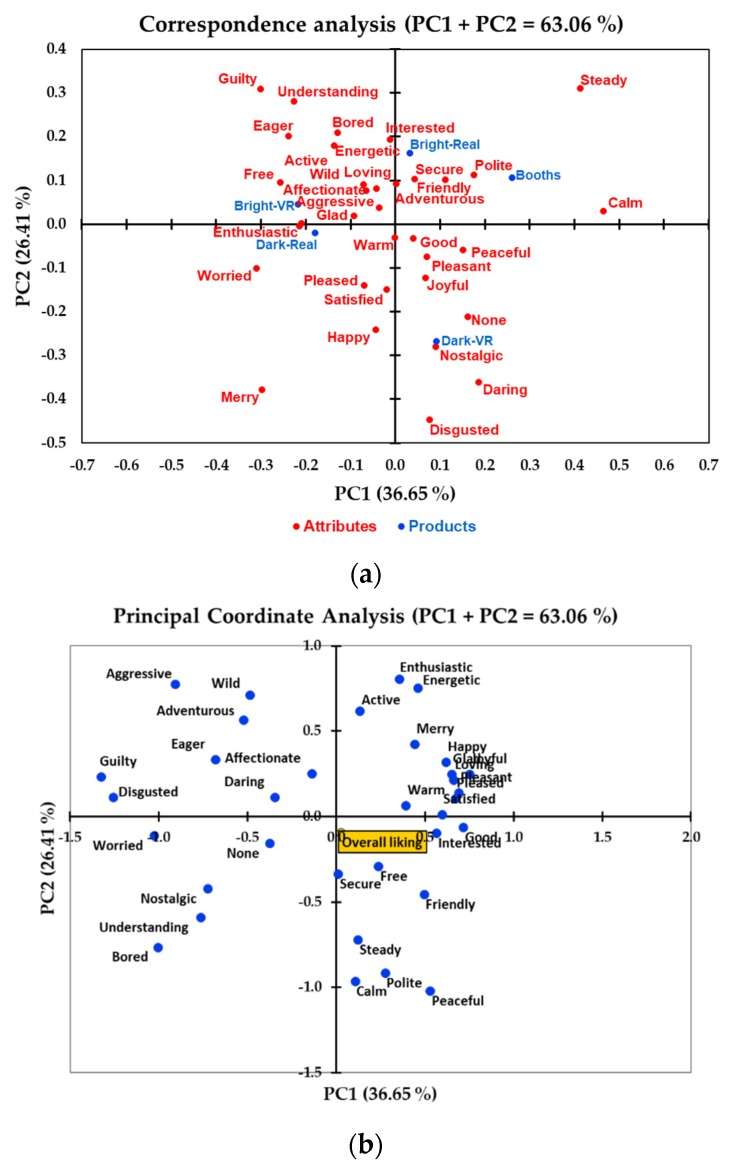
(**a**) Correspondence analysis of the emotion terms for the wine sample in each environment ^1^ and (**b**) principal coordinate analysis of the emotion terms with the overall liking score. ^1^ Booths = traditional sensory booths, Bright-real = bright restaurant real environment, Dark-real = dark restaurant real environment, Bright-VR = bright restaurant VR environment, and Dark-VR = dark restaurant VR environment.

Figure 3a shows the corresponding analysis of the emotional terms of the CATA question related to the wine sample in each environment. The principal component one (PC1) and principal component two (PC2) accounted for 26.41% and 36.65%, respectively, which explained 63.03% of the total data variability. The wine sample was only associated with the emotions “polite” and “calm” under the traditional booths environment. Under the real bright-restaurant environment, participants’ emotions toward the wine sample were associated with “interested”, “secure”, “friendly”, and “loving”.

On the other hand, participants only elicited emotions such as “free” and “enthusiastic” towards the wine sample under the bright-VR environment. The emotions “glad” and “enthusiastic” were associated with the wine under the real dark-restaurant environment. Conversely, “nostalgic”, “daring”, and “disgusted” were associated with the wine under the dark-VR environment. The principal coordinate analysis of the emotion terms concerning the overall liking of the wine sample in different environments is shown in Figure 3b. In general, overall liking was positively associated with the emotion terms “secure”, “free”, “interested”, “good”, and “friendly.” Moreover, the overall liking of the samples was negatively associated with “daring”, “affectionate”, “eager”, “adventurous”, and “wild.”

**Figure 4:**
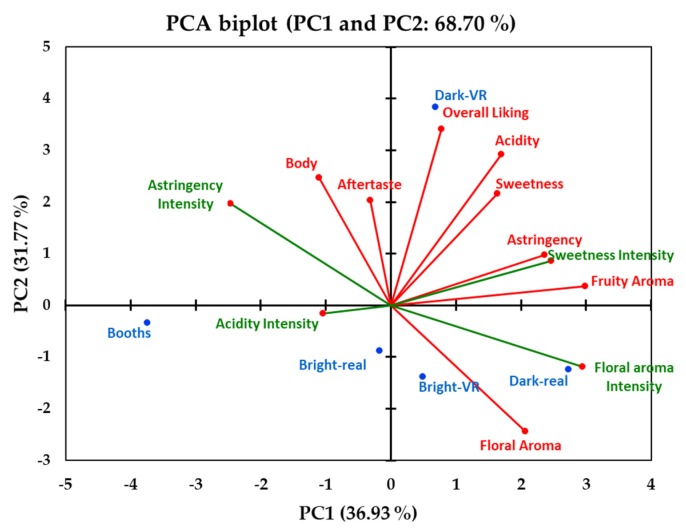
Principal component analysis (PCA) biplot visualizing treatments ^1^ (wine sample in each environment), acceptability (liking; vectors in red color), and intensity attributes (vectors in green color). ^1^ Booths = traditional sensory booths, Bright-real = bright restaurant real environment, Dark-real = dark restaurant real environment, Bright-VR = bright restaurant VR environment, and Dark-VR = dark restaurant VR environment.

The principal component analysis (PCA) biplot shows the acceptability and intensity parameter vectors associated with the five environmental conditions (traditional booths, bright-restaurant, dark-restaurant, bright-VR, and dark-VR) in which the wine sample was tasted (Figure 4). Considering all acceptability and intensity sensory parameters, the PC1 and PC2 accounted for 36.93% and 31.77% of the biplot, respectively, explaining 68.7% of the total data variability. The fruit aroma liking and floral aroma intensity (*factor loading* = 0.89–0.91; data not shown) vectors contributed largely to the discrimination of the environments in the PC1.

On the other hand, the overall liking vector (*factor loadings* = 0.96) contributed largely to the discrimination of the samples in the PC2. According to the PCA, the acidity, sweetness, and aftertaste liking scores were positively associated with overall liking. On the other hand, the liking of the floral aroma was positively associated with the intensity of the floral aroma, but it was negatively associated with the intensity of astringency. The sweetness intensity was positively associated with the liking of the fruity aroma and astringency, but it was negatively associated with the intensity of the acidity. Moreover, the liking of the body was positively associated with the liking of the aftertaste, but it was negatively associated with the liking of the floral aroma. The real dark-restaurant and bright-VR environments were related to higher floral aroma intensity and liking. The dark-VR environment was related to a higher overall liking score, and the traditional booths environment was related to a higher intensity of acidity (Figure 4).

## 4. Discussion

### 4.1. Sensory Responses to the Wine Sample in Different Environments

This study showed that the type of environment (traditional booths, bright-restaurant, dark-restaurant, bright-VR, and dark-VR) had a marginal effect on the sensory acceptability (floral aroma, sweetness, acidity, astringency, body, aftertaste, and overall liking) of the Cabernet Sauvignon wine sample (Table 1 and Table 2). Only the real dark-restaurant environment had a higher acceptability score for the fruity aroma attribute compared to that of the traditional booths (5.99 vs. 5.65, respectively; Table 2). On the other hand, the type of environment significantly affected the intensity perception of the floral aroma and astringency of the wine sample. The wine tasted in both real environments (bright and dark) had a significantly higher floral aroma compared to that of the wine tasted in the booths (8.14–8.61 vs. 7.45, respectively; Table 2). Conversely, the wine tasted in the real dark environment had a significantly lower astringency compared to that of the booths and the dark-VR environment (7.57 vs. 8.34–8.39, respectively; Table 2). 

Virtual reality technology can provide consumers with simulated scenarios that are close to real environments [30]. The present research showed that changing the environment had a significant effect on perception, but that effect was marginal for acceptability. The overall liking of the wine sample in the real environments was similar compared to that of the VR environments. The same effect occurred for the perception of sweetness and acidity; however, there were significant differences between the real and VR in the dark environments for the perception of floral and astringency. Environments may affect consumers’ expectations and experiences of products because their decisions can be unconsciously changed by several extrinsic factors [26]. Ryu and Jang [31] tested different contextual factors such as lighting, facility aesthetics, ambiance music, dining equipment, and employees’ interactions on consumers dining experiences. They found that consumers showed positive emotions to simple environmental changes such as the type of music played and the layout of the dining environment. Moreover, the lighting conditions are very important for the sensory evaluation of foods. Bschaden et al. [32] found that the lighting conditions of the testing environment can affect the saltiness perception of tomato soups. Moreover, consumers tend to choose less healthy food options when the ambient lighting is dim [33]. In the present study, the dark environment might have been the most adequate contextual surrounding for consumers to taste the wine sample, as the perception of fruity and floral aroma had positive effects on consumers. In a similar study, Hersleth et al. [34] found that the wine tasting experience in a reception type of room was significantly improved compared to the tasting of the wines in traditional booths. With the development of more efficient virtual reality technologies, more sensory stimuli can be tested with different contextual situations. The virtual reality technology might potentially replace the use of physical environments in the future, becoming an important tool for sensory evaluation [35].

### 4.2. Emotions and Multivariate Analysis of the Wine Sample in Different Environments

In the present study, changes in the environment affected the elicited consumers’ emotions toward the wine sample. Neutral to positive emotions such as “interested”, “secure”, “polite”, and “friendly” were elicited for the wine tasted in the traditional booths and the real bright restaurant. The wine tasted in the real dark-restaurant and bright-VR environments was associated with “enthusiastic”, “glad”, and “aggressive”. On the other hand, emotions such as “nostalgic” and “daring” were associated with the wine tasted in the dark-VR environment (Figure 3). Berridge and Kringelbach [36] stated that the environmental factors could change the human cognitive ability to elaborate the psychological representation of pleasant events, which might increase the perception of richness and taste by shaping the emotions that are felt toward the stimuli. This means that personal emotions are closely related to the environment in which they occur, and different consumption situations may have a significant effect on consumers’ emotional responses [16]. 

The interaction between product and environment can affect the elicited emotions of consumers during the tasting. Piqueras-Fiszman and Jaeger [37] showed that the contextual scenario is, in fact, a trigger of emotional changes in the consumers. The scenarios that are considered more appropriate to consumers for food consumption had more positive elicited emotions compared to inappropriate contextual environments, which can produce more negative emotional terms associated with the product [37]. In the present study, the use of real restaurant environments triggered more positive elicited emotions compared to that of the traditional booths, which can be an indication of the appropriateness of the restaurant environments when tasting wine samples. In a similar study, Park and Farr [38] showed that consumers’ emotions were affected by the lighting conditions of the testing environment. Changes in mood and emotions can also affect consumers’ taste perception. In the present study, consumers perceived the wine sample to be higher in floral aroma and lower in astringency in the real dark-restaurant environment compared to that in the traditional booths. The real dark-restaurant environment was also responsible for generating positive emotions such as “enthusiastic”, “glad”, and “warm” when tasting the wine sample (Figure 3). Noel and Dando [39] showed that positive emotions were associated with increased sweetness and decreased acidity in ice-cream products, concluding that modulating taste perception can play an important role in emotional eating. In the present study, the overall liking of the wine sample was significantly and positively correlated with the perceived floral aroma and sweetness (*r* = 0.3 to 0.6), and negatively correlated with astringency (*r* = −0.2 to −0.4) for all testing environments (Table S1, Table S2, Table S3, Table S4 and Table S5). 

In the present study, the purchase intent of the wine sample was marginally affected by the change in the environment. Consumers’ decisions to buy a product are affected by several extrinsic factors such as packaging, logo, and color [40,41]. These external factors, combined with the contextual effects of the environment, can greatly modify the purchasing behaviors of consumers. Although traditional laboratory environments are designed to collect data, minimizing the influences of external contextual effects, these environments may lack ecological validity during the tasting. The use of real environments is an option to measure those external factors, but the experimental conditions might produce several variables that are difficult to control [42]. Virtual reality offers a novel solution to measure the effects of environmental factors and having controlled laboratory conditions.

Boesveldt et al. [43] stated that the development of the sensory perception is closely linked with the environment. Sensory perception plays an important role in the acceptance or rejection of food and drinks. Moreover, hedonic responses and preferences can be affected by the familiarity toward the food products that consumers have [28]. However, purchasing decisions of foods can be affected by the surrounding elements of different consumption environments, which means that each product can have a specific consumption environment that is suitable for it [37]. King et al. [44] found that the dining environment greatly affected the consumers’ acceptance and choice of products. García-Segovia et al. [45] stated that the table decoration and dining place also affected consumers’ acceptance and perception of foods. In the present study, the real environment had a greater effect on participants’ perceptions of floral aroma and astringency compared to that of the VR environments. However, the emotions elicited by the wine sample were greatly affected by the VR environments; in particular, this effect was more evident for the dark environment. Future studies are needed to evaluate the effects of other environmental factors such as music and consumers’ interactions with the use of novel virtual reality technologies.

## 5. Limitations

No re-tasting of test samples was possible under the VR testing process, which could be a limitation of this method and the results. Another limitation of this study was that participants were unable to assess the appearance of the sample when wearing the virtual reality headsets. Future technologies such as augmented reality (AR) can overcome this issue and provide a more immersive approach than VR; however, future studies are needed.

## 6. Conclusions

The context and environment affected the perceptual responses of consumers when tasting a wine product. Although liking was marginally affected, the dark-VR environment elicited different emotional responses compared to those of the traditional booths. The virtual reality technology provides a relatively stable and inexpensive method for sensory evaluation by providing a more realistic consuming environment compared to that of traditional sensory booths.

## Figures and Tables

**Table 1:** ANOVA ^1^ table for the acceptability and intensity parameters of the wine by environment samples.

ANOVA Environments								
**Effects ^1^**	**Acceptability**							
	**Floral Aroma**		**Fruity Aroma**		**Sweetness**		**Acidity**	
	***F* Value ^2^**	**Pr > *F*^2^**	***F* Value ^2^**	**Pr > *F*^2^**	***F* Value ^2^**	**Pr > *F*^2^**	***F* Value ^2^**	**Pr > *F*^2^**
Environment	1.15	0.33	1.25	0.29	1.09	0.36	0.92	0.45
Order	0.46	0.50	1.61	0.20	0.00	0.96	0.05	0.83
Environment*Order ^3^	1.44	0.22	2.63	*0.03*	1.66	0.16	0.81	0.52
**Effects ^1^**	**Acceptability**							
	**Mouthfeel Astringency**		**Mouthfeel Body**		**Aftertaste**		**Overall Liking**	
	***F* Value ^2^**	**Pr > *F*^2^**	***F* Value ^2^**	**Pr > *F*^2^**	***F* Value ^2^**	**Pr > *F*^2^**	***F* Value ^2^**	**Pr > *F*^2^**
Environment	0.76	0.55	0.28	0.89	0.16	0.96	0.43	0.79
Order	0.33	0.57	0.68	0.41	2.05	0.15	0.47	0.49
Environment*Order ^3^	0.99	0.41	0.87	0.48	0.85	0.50	1.53	0.19
**Effects ^1^**	**Intensity**							
	**Floral Aroma**		**Sweetness**		**Acidity**		**Astringency**	
	***F* Value ^2^**	**Pr > *F*^2^**	***F* Value ^2^**	**Pr > *F*^2^**	***F* Value ^2^**	**Pr > *F*^2^**	***F* Value ^2^**	**Pr > *F*^2^**
Environment	4.04	*<0.01*	0.73	0.57	1.05	0.38	2.42	*0.05*
Order	0.02	0.89	2.41	0.12	0.47	0.49	0.03	0.86
Environment*Order ^3^	1.42	0.23	1.11	0.35	2.93	*0.02*	5.08	*<0.01*

^1^ ANOVA = analysis of variance (five types of environments (traditional booths, bright-restaurant, dark-restaurant, bright-VR, and dark-VR) and two positional orders. Liking scores were based on a nine-point hedonic scale (1 = dislike extremely, 9 = like extremely). Intensity scores were based on a 15-point Likert scale (1 = absent, 15 = very strong). ^2^ *F* value, mean square/mean square error. Effects were considered significant when the probability Pr > *F* was less than 0.05 (bolded and italicized probabilities). ^3^ The environment effect was crossed with the replicate effect in a two-way factorial design (type of environment by order) using participants as blocks.

**Table 2:** Acceptability and intensity mean values of the wine sample in each environment ^1^.

**Environments ^1^**	**Acceptability ^2^**			
	**Floral Aroma**	**Fruity Aroma**	**Sweetness**	**Acidity**
Traditional booths	5.66 ± 1.59 ^a^	5.65 ± 1.58 ^b^	5.26 ± 1.68 ^a^	5.06 ± 1.71 ^a^
Bright-restaurant	5.90 ± 1.36 ^a^	5.91 ± 1.38 ^a, b^	5.19 ± 1.77 ^a^	5.21 ± 1.78 ^a^
Dark-restaurant	5.94 ± 1.49 ^a^	5.99 ± 1.35 ^a^	5.41 ± 1.66 ^a^	5.23 ± 1.69 ^a^
Bright-VR	5.81 ± 1.32 ^a^	5.79 ± 1.31 ^a, b^	5.47 ± 1.72 ^a^	5.10 ± 1.65 ^a^
Dark-VR	5.65 ± 1.49 ^a^	5.90 ± 1.44 ^a, b^	5.59 ± 1.77 ^a^	5.44 ± 1.66 ^a^
**Environments ^1^**	**Acceptability ^2^**			
	**Astringency**	**Body**	**Aftertaste**	**Overall Liking**
Traditional booths	5.15 ± 1.81 ^a^	5.67 ± 1.47 ^a^	5.59 ± 1.49 ^a^	5.57 ± 1.60 ^a^
Bright-restaurant	5.48 ± 1.54 ^a^	5.73 ± 1.36 ^a^	5.49 ± 1.37 ^a^	5.58 ± 1.46 ^a^
Dark-restaurant	5.41 ± 1.85 ^a^	5.58 ± 1.57 ^a^	5.58 ± 1.55 ^a^	5.60 ± 1.66 ^a^
Bright-VR	5.28 ± 1.43 ^a^	5.62 ± 1.31 ^a^	5.50 ± 1.50 ^a^	5.54 ± 1.52 ^a^
Dark-VR	5.44 ± 1.79 ^a^	5.75 ± 1.54 ^a^	5.60 ± 1.59 ^a^	5.77 ± 1.56 ^a^
**Environments ^1^**	**Intensity ^2^**			
	**Floral Aroma**	**Sweetness**	**Acidity**	**Astringency**
Traditional booths	7.45 ± 3.04 ^c^	6.62 ± 2.88 ^a^	8.16 ± 2.67 ^a^	8.39 ± 2.14 ^a^
Bright-restaurant	8.14 ± 3.13 ^a, b^	6.67 ± 2.76 ^a^	8.25 ± 2.84 ^a^	8.23 ± 2.55 ^a^
Dark-restaurant	8.61 ± 2.93 ^a^	6.97 ± 2.71 ^a^	8.09 ± 2.44 ^a^	7.57 ± 2.62 ^b^
Bright-VR	7.96 ± 2.65 ^b, c^	7.05 ± 3.00 ^a^	7.65 ± 2.57 ^a^	7.78 ± 2.79 ^a, b^
Dark-VR	7.84 ± 2.84 ^b, c^	7.01 ± 2.93 ^a^	7.94 ± 2.80 ^a^	8.34 ± 2.59 ^a^

^1^ Five environments were tested (traditional booths, bright-restaurant, dark-restaurant, bright-VR, and dark-VR). Means and standard deviations of 53 participants. ^2^ Liking scores were based on a nine-point hedonic scale (1 = dislike extremely, 9 = like extremely). Intensity scores were based on a 15-point Likert scale (1 = absent, 15 = very strong). ^a–c^ Means with different superscripts in each column within each attribute indicate significant differences (*p* < 0.05) by the Tukey studentized range Honest Significant Difference (HSD) test.

**Table 3:** Penalty analysis results for the sweetness, acidity, astringency, and body of the wine sample in different environments ^1^.

Environment	Sweetness		Acidity		Astringency		Body	
	Too Little	Too Much	Too Little	Too Much	Too Little	Too Much	Too Little	Too Much
Booths	***1.54***	−0.25	1.66	***1.44***	0.14	***1.52***	0.72	0.17
Bright-real	***1.25***	0.21	0.43	***1.30***	0.01	***1.34***	−0.20	−0.67
Dark-real	***1.99***	0.06	1.58	***1.94***	0.31	***1.94***	−0.08	−0.02
Bright-VR	***1.51***	0.60	0.89	***1.17***	1.47	***1.76***	0.66	0.01
Dark-VR	0.40	−1.64	−0.47	***1.24***	−1.06	***1.02***	−1.35	1.00

^1^ Booths = traditional sensory booths, Bright-real = bright restaurant real environment, Dark-real = dark restaurant real environment, Bright-VR = bright restaurant VR environment, and Dark-VR = dark restaurant VR environment. Values represent the mean drops using the nine-point hedonic scale. Mean drops were considered significant when the probability Pr > *F* was less than 0.05 (bolded and italicized values).

## Supplementary Materials

The following are available online at https://www.mdpi.com/2304-8158/9/2/191/s1, Table S1: Correlation analysis (r)* of the variables measured for the wine sample in the traditional booth environment, Table S2: Correlation analysis (r)* of the variables measured for the wine sample in the bright restaurant environment, Table S3: Correlation analysis (r)* of the variables measured for the wine sample in the dark restaurant environment, Table S4: Correlation analysis (r)* of the variables measured for the wine sample in the bright VR environment, Table S5: Correlation analysis (r)* of the variables measured for the wine sample in the dark VR environment.
