# Supplementary material for: Effects of Context and Virtual Reality Environments on the Wine Tasting Experience, Acceptability, and Emotional Responses of Consumers
Source: Foods. 2020 Feb 14;9(2):191. doi: 10.3390/foods9020191 (PMC7073756; doi:10.3390/foods9020191)
Supplement: Supplementary file 1 [file foods-09-00191-s001.pdf]

Table S1 Correlation analysis (r)* of the variables measured for the wine sample in the traditional booth environment

Variables	**Acceptability**								**Intensity**			
	**Floral Aroma**	**Fruit Aroma**	**Sweetness**	**Acidity**	**Astringent**	**Body**	**Aftertaste**	**Overall liking**	**Floral aroma**	**Sweetness**	**Acidity**	**Astringent**
Floral Aroma	**1.00**	**0.73**	**0.44**	0.17	0.15	**0.20**	**0.26**	**0.43**	**0.62**	**0.38**	0.02	−0.16
Fruit Aroma		**1.00**	**0.54**	**0.27**	**0.20**	**0.30**	**0.41**	**0.51**	**0.49**	**0.51**	−0.05	−0.13
Sweetness			**1.00**	**0.52**	**0.40**	**0.44**	**0.53**	**0.75**	**0.37**	**0.56**	−0.06	**−0.29**
Acidity				**1.00**	**0.65**	**0.43**	**0.41**	**0.57**	0.08	**0.32**	−0.19	−0.12
Astringent					**1.00**	**0.64**	**0.47**	**0.61**	**0.19**	**0.27**	−0.16	**−0.33**
Body						**1.00**	**0.62**	**0.67**	**0.25**	**0.31**	−0.10	**−0.25**
Aftertaste							**1.00**	**0.77**	**0.28**	**0.40**	−0.07	**−0.30**
Overall liking								**1.00**	**0.39**	**0.59**	−0.19	**−0.41**
Floral aroma									**1.00**	**0.64**	0.07	**−0.25**
Sweetness										**1.00**	−0.07	**−0.43**
Acidity											**1.00**	**0.36**
Astringency												**1.00**

*Pearson correlation analysis.

Values in bold are different from 0 with a significance level alpha=0.05

Table S2 Correlation analysis (r)* of the variables measured for the wine sample in the bright restaurant environment

**Variables**	**Acceptability**								**Intensity**			
	**Floral Aroma**	**Fruit Aroma**	**Sweetness**	**Acidity**	**Astringent**	**Body**	**Aftertaste**	**Overall liking**	**Floral aroma**	**Sweetness**	**Acidity**	**Astringent**
Floral Aroma	**1.00**	**0.67**	**0.28**	**0.36**	**0.40**	**0.39**	**0.33**	**0.37**	**0.42**	0.19	**−0.19**	−0.15
Fruit Aroma		**1.00**	**0.35**	**0.27**	**0.30**	**0.35**	**0.35**	**0.35**	**0.29**	0.15	**−0.24**	**−0.20**
Sweetness			**1.00**	**0.63**	**0.53**	**0.54**	**0.62**	**0.68**	0.16	**0.58**	**−0.42**	**−0.41**
Acidity				**1.00**	**0.60**	**0.57**	**0.58**	**0.64**	0.07	**0.33**	**−0.30**	**−0.30**
Astringent					**1.00**	**0.59**	**0.55**	**0.64**	**0.20**	**0.45**	**−0.19**	**−0.42**
Body						**1.00**	**0.59**	**0.69**	0.14	**0.29**	**−0.27**	**−0.37**
Aftertaste							**1.00**	**0.82**	**0.27**	**0.46**	**−0.37**	**−0.38**
Overall liking								**1.00**	**0.34**	**0.52**	**−0.40**	**−0.41**
Floral aroma									**1.00**	**0.43**	0.11	−0.03
Sweetness										**1.00**	−0.14	−0.15
Acidity											**1.00**	**0.33**
Astringency												**1.00**

*Pearson correlation analysis.

Values in bold are different from 0 with a significance level alpha=0.05

Table S3 Correlation analysis (r)* of the variables measured for the wine sample in the dark restaurant environment

**Variables**	**Acceptability**								**Intensity**			
	**Floral Aroma**	**Fruit Aroma**	**Sweetness**	**Acidity**	**Astringent**	**Body**	**Aftertaste**	**Overall liking**	**Floral aroma**	**Sweetness**	**Acidity**	**Astringent**
Floral Aroma	**1.00**	**0.66**	**0.48**	**0.41**	**0.36**	**0.48**	**0.50**	**0.54**	**0.52**	**0.33**	**−0.29**	−0.12
Fruit Aroma		**1.00**	**0.42**	**0.28**	**0.36**	**0.44**	**0.53**	**0.55**	**0.48**	**0.33**	**−0.23**	−0.13
Sweetness			**1.00**	**0.69**	**0.62**	**0.61**	**0.68**	**0.75**	0.12	**0.56**	**−0.47**	**−0.36**
Acidity				**1.00**	**0.65**	**0.54**	**0.57**	**0.66**	0.12	**0.36**	**−0.38**	**−0.21**
Astringent					**1.00**	**0.72**	**0.71**	**0.78**	**0.22**	**0.47**	**−0.27**	**−0.41**
Body						**1.00**	**0.70**	**0.78**	**0.34**	**0.43**	**−0.30**	**−0.28**
Aftertaste							**1.00**	**0.87**	**0.38**	**0.54**	**−0.34**	**−0.23**
Overall liking								**1.00**	**0.43**	**0.62**	**−0.37**	**−0.30**
Floral aroma									**1.00**	**0.40**	−0.01	0.14
Sweetness										**1.00**	−0.16	**−0.20**
Acidity											**1.00**	**0.39**
Astringency												**1.00**

*Pearson correlation analysis.

Values in bold are different from 0 with a significance level alpha=0.05

Table S4 Correlation analysis (r)* of the variables measured for the wine sample in the bright VR environment

**Variables**	**Acceptability**								**Intensity**			
	**Floral Aroma**	**Fruit Aroma**	**Sweetness**	**Acidity**	**Astringent**	**Body**	**Aftertaste**	**Overall liking**	**Floral aroma**	**Sweetness**	**Acidity**	**Astringent**
Floral Aroma	**1.00**	**0.69**	**0.54**	**0.31**	**0.32**	**0.34**	**0.44**	**0.55**	**0.54**	**0.51**	−0.08	0.00
Fruit Aroma		**1.00**	**0.56**	**0.28**	**0.30**	**0.27**	**0.46**	**0.55**	**0.48**	**0.51**	−0.14	**−0.19**
Sweetness			**1.00**	**0.56**	**0.36**	**0.39**	**0.51**	**0.57**	**0.37**	**0.81**	**−0.30**	**−0.30**
Acidity				**1.00**	**0.56**	**0.53**	**0.43**	**0.51**	0.16	**0.49**	**−0.22**	**−0.25**
Astringent					**1.00**	**0.63**	**0.54**	**0.54**	**0.26**	**0.30**	−0.12	**−0.21**
Body						**1.00**	**0.62**	**0.66**	0.14	**0.34**	−0.01	−0.16
Aftertaste							**1.00**	**0.85**	**0.27**	**0.40**	−0.10	**−0.19**
Overall liking								**1.00**	**0.31**	**0.50**	−0.14	−0.17
Floral aroma									**1.00**	**0.47**	−0.01	−0.02
Sweetness										**1.00**	**−0.31**	**−0.34**
Acidity											**1.00**	**0.45**
Astringency												**1.00**

*Pearson correlation analysis.

Values in bold are different from 0 with a significance level alpha=0.05

Table S5 Correlation analysis (r)* of the variables measured for the wine sample in the dark VR environment

Variables	Acceptability								Intensity			
	**Floral Aroma**	**Fruit Aroma**	**Sweetness**	**Acidity**	**Astringent**	**Body**	**Aftertaste**	**Overall liking**	**Floral aroma**	**Sweetness**	**Acidity**	**Astringent**
Floral Aroma	**1.00**	**0.78**	**0.55**	**0.35**	**0.35**	**0.50**	**0.53**	**0.54**	**0.53**	**0.50**	**−0.26**	−0.13
Fruit Aroma		**1.00**	**0.62**	**0.31**	**0.27**	**0.44**	**0.51**	**0.57**	**0.56**	**0.54**	**−0.23**	−0.14
Sweetness			**1.00**	**0.49**	**0.38**	**0.56**	**0.61**	**0.70**	**0.34**	**0.59**	**−0.34**	**−0.34**
Acidity				**1.00**	**0.74**	**0.62**	**0.47**	**0.65**	**0.19**	**0.38**	**−0.32**	**−0.25**
Astringent					**1.00**	**0.61**	**0.51**	**0.57**	**0.30**	**0.37**	**−0.19**	**−0.26**
Body						**1.00**	**0.57**	**0.66**	**0.31**	**0.33**	**−0.26**	**−0.26**
Aftertaste							**1.00**	**0.85**	**0.35**	**0.53**	−0.16	**−0.26**
Overall liking								**1.00**	**0.39**	**0.59**	**−0.31**	**−0.39**
Floral aroma									**1.00**	**0.56**	−0.03	−0.10
Sweetness										**1.00**	**−0.37**	**−0.26**
Acidity											**1.00**	**0.47**
Astringency												**1.00**

*Pearson correlation analysis.

Values in bold are different from 0 with a significance level alpha=0.05
